# Isolation and Characterization of EstC, a New Cold-Active Esterase from *Streptomyces coelicolor* A3(2)

**DOI:** 10.1371/journal.pone.0032041

**Published:** 2012-03-02

**Authors:** Guillaume Brault, François Shareck, Yves Hurtubise, François Lépine, Nicolas Doucet

**Affiliations:** 1 INRS-Institut Armand-Frappier, Université du Québec, Laval, Québec, Canada; 2 Innu-Science Canada, Inc., Trois-Rivières, Québec, Canada; Auburn University, United States of America

## Abstract

The genome sequence of *Streptomyces coelicolor* A3(2) contains more than 50 genes coding for putative lipolytic enzymes. Many studies have shown the capacity of this actinomycete to store important reserves of intracellular triacylglycerols in nutrient depletion situations. In the present study, we used genome mining of *S. coelicolor* to identify genes coding for putative, non-secreted esterases/lipases. Two genes were cloned and successfully overexpressed in *E. coli* as His-tagged fusion proteins. One of the recombinant enzymes, EstC, showed interesting cold-active esterase activity with a strong potential for the production of valuable esters. The purified enzyme displayed optimal activity at 35°C and was cold-active with retention of 25% relative activity at 10°C. Its optimal pH was 8.5–9 but the enzyme kept more than 75% of its maximal activity between pH 7.5 and 10. EstC also showed remarkable tolerance over a wide range of pH values, retaining almost full residual activity between pH 6–11. The enzyme was active toward short-chain *p*-nitrophenyl esters (C2–C12), displaying optimal activity with the valerate (C5) ester (*k*_cat_/*K*_m_ = 737±77 s^−1^ mM^−1^). The enzyme was also very active toward short chain triglycerides such as triacetin (C2:0) and tributyrin (C4:0), in addition to showing good primary alcohol and organic solvent tolerance, suggesting it could function as an interesting candidate for organic synthesis of short-chain esters such as flavors.

## Introduction

Carboxylesterases (EC 3.1.1.1) and lipases (triacylglycerol hydrolases; EC 3.1.1.3) are important industrial enzymes with numerous applications in biotechnology (reviewed in [1]). Esterases hydrolyze water-soluble or emulsified esters with relatively short fatty acid chains (<than 10 carbons) while “true” lipases are generally more active toward emulsified, long-chain fatty acids [2], [3]. These enzymes, which catalyze reactions in a regio- and/or enantioselective fashion [4], can catalyze the release of free fatty acids from triglycerides and under certain conditions can also perform esterification and transesterification reactions. Furthermore, these biocatalysts are generally stable in organic solvents, allowing their use in numerous industrial applications [5]. Although lipolytic enzymes are found in all living organisms, most of the commercial enzymes originate from microbial sources. As a result, bacterial genome mining of versatile microorganisms offers an attractive opportunity to uncover new lipolytic biocatalysts displaying interesting biochemical properties.

**Figure 1:**
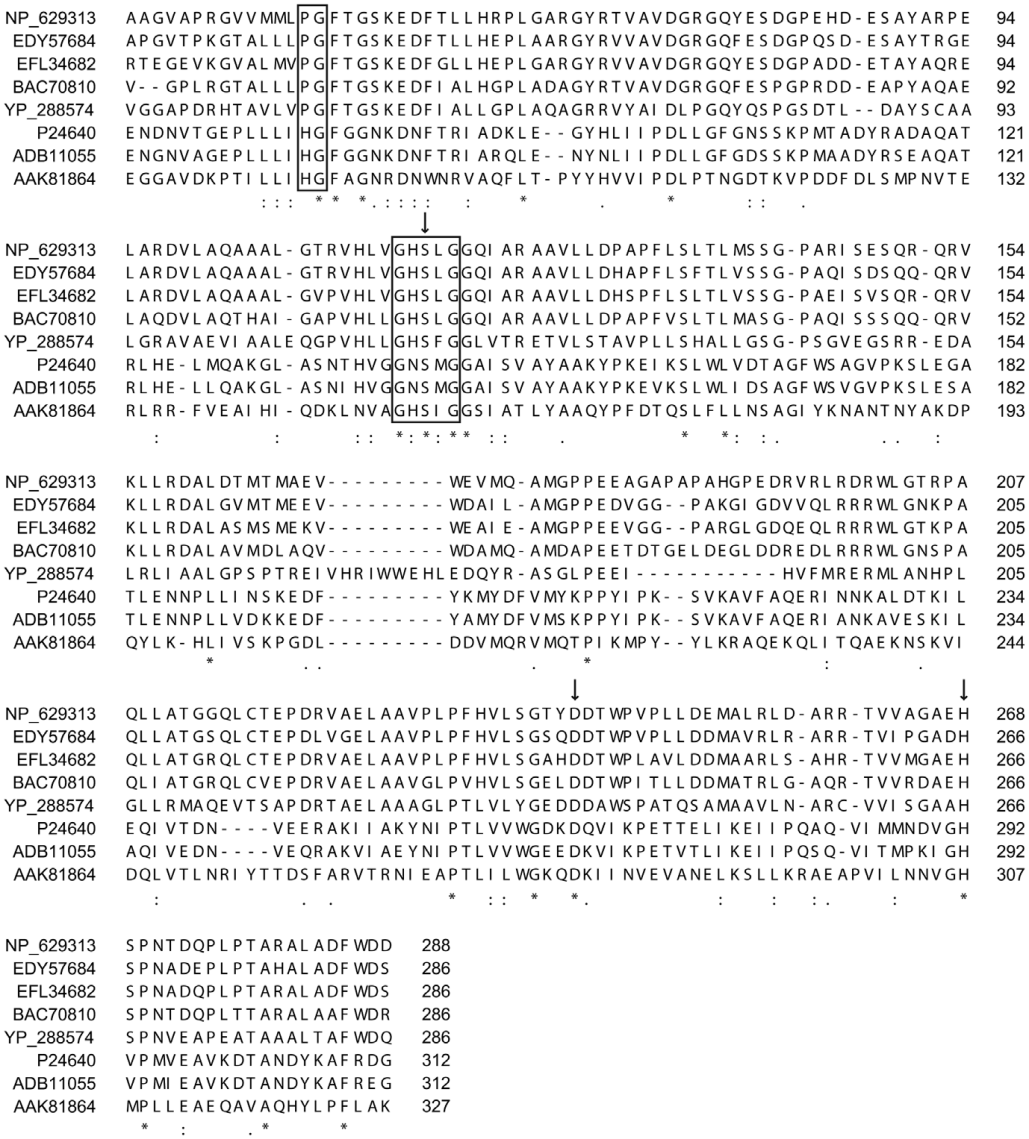
Sequence alignment of EstC with homologous lipases. NP_629313, EstC from *S. coelicolor* A3(2); EDY57684, putative hydrolase from *S. sviceus* ATCC 29083; EFL34682, putative hydrolase from *S. viridochromogenes* DSM 40736; BAC70810, putative hydrolase from *S. avermitilis* MA-4680; YP_288574, putative hydrolase from *Thermobifida fusca* strain YX; P24640, lipase 3 from *Moraxella* sp.; ADB11055, lipase from *Psychrobacter* sp. G; AAK81864, lipase from *Streptococcus* sp. (N1). The conserved Gly-X_aa_-Ser-X_aa_-Gly pentapeptide and the proposed oxyanion hole are boxed. Residues of the catalytic triad are shown by arrows. Sequence alignment was performed with TCoffee [52], [53].

Bacterial lipases and esterases are grouped into 8 different families based on their sequence, structure and biological function [6]. In all cases, these enzymes are characterized by a typical catalytic triad formed of a nucleophilic serine, a catalytic acid (aspartate or glutamate) and a histidine residue, all of which appear in this order in the protein sequence (Figure 1) [6]. The catalytic serine is typically located among a conserved Gly-X_aa_-Ser-X_aa_-Gly pentapeptide which forms a sharp elbow in the center of the canonical α/β-fold [3]. Many reports suggest that true lipases are distinguished from esterases by harboring a lid domain that covers the hydrophobic catalytic cleft [7], [8]. This lid moves to expose the catalytic cleft at the lipid-water interface following an activation mechanism typical of lipases. However, numerous reports reveal exceptions to this rule, such as the well-studied *Candida antarctica* Lip B [6], [7], [9].

Streptomycetes are ubiquitous soil-dwelling saprophyte bacteria that can feed on diverse carbon sources available in the soil biotope. These Gram-positive, filamentous bacteria are best known as producers of numerous antibiotics as well as various extracellular hydrolytic enzymes [10], [11]. As a result, it is not surprising that ubiquitous lipolytic activity can be found among streptomycetes [10]–[14]. Some reports also show that streptomycetes such as *Streptomyces coelicolor* and the closely related specie *Streptomyces lividans* are capable of important accumulations of cytoplasmic triacylglycerols (TAGs) in the stationary phase [15]–[18]. It was presumed that the build-up of TAGs could serve as a carbon source for the synthesis of polyketide antibiotics in nutrient depletion situations [15], [19], [20]. However, recent findings suggest that the storage of TAGs could simply be used as an energy reserve [16]. Nevertheless, streptomycetes differ from most lipid accumulating bacteria, which generally synthesize specialized polyesters such as poly(3-hydroxybutyric acid) (poly(3HB)) or other polyhydroxyalkanoic acids (PHA) [17]. While the accumulation of TAGs is a common feature among actinomycetes, streptomycetes differ in the accumulation of neutral lipids of rare types, such as *iso-* and *anteiso-*ramified TAGs with odd carbon chains (13, 15 and 17 carbons) [17], [19]. Furthermore, this accumulation of TAGs was recently correlated to the activation of lipolytic activity [15], [18], but the characterization of lipolytic enzymes implicated in such activity remains elusive [17], [21].

The genome of *Streptomyces coelicolor* A3(2) was the first member of the streptomycetes to be fully sequenced [22]. Genome annotation of this organism showed that at least 50 open reading frames (ORFs) encode for putative lipolytic enzymes [23], five of which have been characterized so far: SCO0713 (*lip*A), SCO1725, SCO7513, SCO7131 (*est*A), and SCO6966 (*est*B) [21], [23]–[25]. In addition to being cold-active and thermostable, these enzymes show broad substrate specificities for TAGs and aryl-esters. However, previous reports mainly focused on extracellular enzymes of *S. coelicolor*. Because this organism can store important reserves of odd-carbon chains, ramified TAGs, characterization of intracellular putative lipolytic biocatalysts might result in the uncovering of enzymes with interesting substrate specificities. In the present study, we used genome mining of *S. coelicolor* to identify genes encoding putative, non-secreted esterases/lipases. Two genes were cloned and successfully overexpressed in *E. coli* as His-tagged fusion proteins. One of the recombinant enzymes, EstC, showed interesting cold-active esterase activity with a strong potential for the production of valuable esters. The enzymatic characterization of EstC is herein presented in comparison with its homologue EstB and several other cold-active lipolytic enzymes of the same family. Our results suggest that EstC demonstrates unique features that deserve further consideration for cold-adapted industrial processes.

## Materials and Methods

### Bacterial strains and plasmids

The strain *Escherichia coli* BL21 (DE3) was employed for all subcloning and heterologous expression of selected genes. Expression vector pET16b (EMD Chemicals) was used for the production of the N-terminal histidine-tagged recombinant proteins. Genomic DNA employed for gene amplification was extracted from *Streptomyces coelicolor* M145 following standard procedures [26].

### DNA manipulations

From the available annotated genome sequence [22], genes coding for putative lipolytic enzymes without a secretion signal peptide were selected for subcloning (Table S1). Sequences of the annotated putative genes were analyzed using the SignalP 3.0 server (www.cbs.dtu.dk/services/SignalP/). Genes without a Gram-positive secretion peptide signal were selected for subsequent amplification by PCR. Selected genes were *est*B (SC6F7.19c, locus SCO6966) and *est*C (SCP8.28c, locus SCO5165). The recombinant genes were PCR-amplified using the following primers: 6F7.19c-5′ (5′-AAACATATGGCCGAGGCCCGCGAGCACACG-3′), 6F7.19c-3′ (5′-ATTCTCGAGTCAGCGGGCGAGCACGCCGTC-3′) for the *est*B gene, and SCP8_NdeI (5′-AAACATATGGTGAGCAGGAACGCCGCCTTC-3′), SCP8_XhoI (5′- TTTCTCGAGTCAGCGCACGTACG-3′) for the *est*C gene. Restriction sites were added to both ends (5′-*Nde*I and 3′-*Xho*I, both underlined) to allow subcloning in vector pET16b. PCR-amplified fragments were cleaned using Qiagen PCR cleaning kit, cloned into the *Nde*I/*Xho*I-digested pET16b, and transformed into *E. coli* BL21 (DE3), yielding the pET16b-EstB and pET16b-EstC constructs used for protein expression. The integrity of both mutant plasmid strands was confirmed by DNA sequencing using forward and reverse T7 universal primers (Genome Quebec).

### Heterologous expression and purification

For heterologous expression, 100 mL of Luria-Bertani (LB) medium containing 100 µg/mL of carbenicillin was inoculated from a fresh Petri dish. The liquid culture was incubated at 37°C and 240 rpm until it reached an A_600_ of 0.6–0.8, after which it was cooled on ice and induced by the addition of 0.25 mM IPTG. The culture was further incubated 24 h under agitation (240 rpm) at 16°C. Cells were harvested, washed with 50 mM bicine buffer pH 7.0 and the cell pellet was resuspended in Q-Proteome lysis buffer (Qiagen). To ensure complete lysis, cells were further disrupted with 250 mg/mL of 0.1 mm silica beads employing a Fast-Prep bead-beater (Biospec). Cells were shaken at 6 m/s for two bursts of 45 seconds. Lysed cells were centrifuged for 15 min at 15 000 *g* and 4°C to remove cell debris. The soluble fraction was filtered on a 0.22 µm membrane, diluted with 1 volume of binding buffer (100 mM bicine pH 8.3, 600 mM NaCl, and 20 mM imidazole), and loaded on a Ni-NTA column (Qiagen). The column contained 3 mL of agarose beads in a Fastprep holder (Bio-Rad) and binding was done by incubation for 60 min at 4°C. The column was then washed with 5 column volumes of 50 mM bicine pH 8.3, 300 mM NaCl, 50 mM imidazole, and the protein was eluted with 5 column volumes of the same buffer containing 250 mM imidazole. Fractions of 2 mL were collected and analyzed by SDS-PAGE and Western blot. Fractions containing the recombinant enzymes were desalted in 50 mM bicine buffer pH 8.5 by ultrafiltration using a 3 kDa Amicon membrane. Protein concentration was determined by a BCA kit (Thermo Scientific) following the manufacturer's instructions.

### SDS-PAGE and Western blot

Fractions obtained from chromatography were analyzed using standard SDS-PAGE procedures with commercial precast 12% RunBlue® gels (Expedeon). Gels were stained with Rapid Stain (Expedeon). Transblot was done over a PVDF membrane in cold transfer buffer (Tris 12 mM, glycine 96 mM, 20% (v/v) methanol). Recombinant His-tagged proteins were revealed with a 1/5000 dilution of anti-penta His-HRP monoclonal antibody (Qiagen) in the kit blocking buffer. Bands were revealed using 1 mM *o*-dianisidine in a revealing solution (100 mM KH_2_PO_4_, 100 mM (NH_4_)_2_SO_4_, 0.5% H_2_O_2_ (v/v)).

### Zymogram analysis

In-gel enzymatic activity was performed according to a previously described SDS-PAGE procedure [25] with the following modifications. Proteins were heated at 70°C for 10 min in a Laemmli sample buffer without dithiotreitol (DTT) or β-mercaptoethanol, and loaded on a 12% Expedeon gel. To remove SDS, the gel was washed twice for 15 minutes in a refolding buffer (20 mM Tris-HCl pH 8.8, 2% casein (w/v)) with gentle agitation, and further incubated 30 min in 20 mM Tris-HCl pH 8.8, 2% Triton X-100 (v/v). Gels were finally washed twice with deionized water to remove Triton X-100. The lipolytic activity was visualized under UV illumination after a 5-minute incubation in a 20 mM Tris-HCl pH 8.0 solution containing 100 µM of 4-methyl-umbelliferyl (MUF)-butyrate [27].

### Mass spectrometry

The mass spectrum of the whole protein was obtained with a Quattro Premier XE (Waters) by infusion of a 10% acetic acid aqueous solution of the purified protein and using a scan range of m/z 700 to 1100. Deconvolution of the resulting mass spectra was performed using the MaxEnt1 software (Micromass). For peptide sequencing, stained SDS-PAGE bands were excised and treated as mentioned previously [25]. The hydrolyzed peptides were analyzed using a Q-Trap hybrid linear ion trap triple-quadrupole mass spectrometer (Applied Biosystems) coupled to an Agilent 1100 Nanopump equipped with a 75 µm×150 mm Zorbax 300SB C_18_ column (Agilent). Accumulation of MS-MS data was performed with the Analyst Software version 1.4 (Applied Biosystems). Mascot (Matrix Science) was used to create peak lists from MS and MS-MS raw data.

### Enzyme assays

#### Standard assays and enzyme kinetics

Standard assays were done by measuring the rate of hydrolysis of different *p*-nitro-phenyl saturated fatty acid esters (*p*NP-C2:0 to *p*NP-C18:0). Substrates were prepared as 50 mM stocks in HPLC-grade 2-propanol and conserved at −80°C. All reactions were performed in triplicates using standard 96-well microplates. Each well contained 0.2 mL of reaction buffer (50 mM Tris-HCl pH 8.5, 0.1% Triton X-100 (v/v) and 0.5 mM of each substrate), and the reactions were started by the addition of 0.02 mL of enzyme diluted at 0.0125 mg/mL. Reactions were monitored every 30 seconds for 5 minutes at 405 nm and 25°C employing a thermostated Varioskan microplate reader (Thermo Electron Corp.).

Enzyme kinetic assays were done using short-chain *p*NP-esters (C2, C4, C5, C6 and C8) at concentrations varying from 0.025 to 6.0 mM in the aforementioned assay buffer at the optimal temperature of 35°C. One unit (U) was defined as the amount of enzyme needed to release 1 µmol of *p*NP per minute. Initial velocity versus substrate concentration data was fitted to a non-linear regression transformation using the GraphPad Prism 3.0 software. The catalytic rate constant *k*_cat_ (s^−1^) was calculated from the initial velocity according to the equation *k*_cat_ = V_max_/[E]. Assays with triglycerides were performed using a pH stat method employing a Metrohm 800 Dosino coupled to a thermostatic circulating water bath. Briefly, the fatty acids released from glyceryl triacetin (C2:0) and glyceryl tributyrin (C4:0) were automatically titrated using a 0.05N NaOH solution to maintain a constant pH of 8.5 at 35°C in a jacketed vessel. Stable emulsions of each substrate were prepared in 5 mM Tris-HCl pH 8.5 containing 1% (w/v) arabic gum using a kitchen mixer (Magic Bullet®) for 2 minutes at 3000 rpm. Reactions were performed in 29 mL of each substrate solutions and were started by the addition of 1 mL of the various enzyme dilutions under vigorous agitation. The release of fatty acids was automatically titrated using the Tiamo software version 1.3 and the activity was measured as microequivalents (μeq) of NaOH necessary to neutralize the free fatty acid released from the enzymatic reaction in 1 minute. One unit (U) was defined as the amount of enzyme releasing 1 µmol of fatty acid per minute in the conditions described.

#### Effect of pH on enzyme activity

Determination of enzyme activity at different pH values was performed in 40 mM sodium acetate, 50 mM MES, 50 mM glycine, 40 mM Tris buffer with *p*NP-C8 as substrate to minimize spontaneous hydrolysis observed at higher pH. Reactions were run for 5 min at 25°C and subsequently quenched with 10% (v/v) of 3N HCl. The pH was then titrated back to 8.5 and the released *p*-nitrophenol (*p*NP) was measured at 405 nm. Impact of enzyme pre-incubation at different pH values was performed by incubating 5 µg of enzyme in 0.1 mL of 40 mM sodium acetate, 50 mM MES, 50 mM glycine, 40 mM Tris buffer at 25°C for one hour. The enzyme was then diluted 4-fold and used in the same assay conditions as previously mentioned with *p*NP-C5 as substrate.

#### Effect of temperature on enzyme activity

The effect of temperature on enzyme activity was determined by assaying the hydrolytic activity towards *p*NP-C8 in 1 mL cuvettes using a thermo-controlled Lambda 25 UV/Vis spectrophotometer (Perkin Elmer) coupled to an Isotemp 3016D water bath (Fisher Scientific). Thermostability assays were done by incubating 5 µg of enzyme in a 50 mM Tris-HCl pH 8.5 buffer at various temperatures for 1 hour before being cooled on ice. Residual activity was measured as mentioned above.

#### Effect of ions, agents, and organic solvents on enzyme activity

Effect of various ions, agents, and organic solvents on enzyme activity was determined by pre-incubating 5 µg of the purified enzyme for one hour in 50 mM Tris-HCl pH 8.5 at 25°C. Water-miscible solvent concentrations tested were 10 and 30% (v/v). After a 1-hour pre-incubation, the enzyme was diluted 4-fold to ensure minimal carry-over for the standard assay. Residual activity toward *p*NP-C5 was compared to that of the enzyme incubated in a 50 mM Tris-HCl pH 8.5 buffer at 25°C without any organic solvent.

## Results and Discussion

Lipolytic enzymes are important biotechnological tools for various industrial applications. Lately, enzymes from psychrophilic microorganisms have attracted much attention because of their high catalytic activity at low temperatures and their inherently broad substrate specificity relative to their thermophilic counterparts [28]. These properties allow the use of cold-active lipases and carboxylesterases in numerous applications of the detergent and dairy industries, in addition to the organic synthesis of fragile pharmaceutical compounds [28]. Therefore, a great deal of effort has been devoted to the discovery and characterization of new lipolytic enzymes from various psychrophilic microorganisms [29]–[36]. Genome mining of versatile mesophilic microbes such as *S. coelicolor* has also proven to be a successful source of cold-adapted lipolytic enzymes [23]. Also, *S. coelicolor* was shown to accumulate important amounts of cytoplasmic TAGs during the stationary growth. While build-up of TAGs is common among eukaryotes and some prokaryotes, the saturated, odd-numbered carbon chain TAGs of *S. coelicolor* are unique in composition [16]. Their metabolism is associated with inherent but yet uncharacterized lipolytic activity [15], [18]. Herein we focus on the characterization of the intracellular esterase EstC from *S. coelicolor*, an enzyme displaying promising activity for cold-adapted industrial processes.

Genome mining of *S. coelicolor* predicted the existence of more than 50 putative lipolytic enzymes [23]. We analyzed all sequences with the SignalP server (www.cbs.dtu.dk/services/SignalP/) and surveyed the putative hydrolases for the absence of a Gram-positive secretion signal peptide. This allowed the identification of seven genes as possible cytoplasmic enzymes (Table S1). All seven genes were cloned in the pET16b vector but only SCO5165, SCO6966 and SCO7131 were successfully overexpressed as soluble proteins. Since two of those genes had already been fully characterized as esterases (SCO7131 (*est*A) and SCO6966 (*est*B)) [23], [24]), we focused on the third member of this gene family (SCO5165). Preliminary enzyme characterization showed that the protein product of SCO5165 is only active toward MUF-butyrate and displays no activity toward MUF-palmitate and MUF-oleate, suggesting carboxylesterase specificity (results not shown). In accordance with the previously characterized esterases of *S. coelicolor* [23], [24], the protein product of SCO5165 was named EstC.

Alignment of EstC amino acid sequence with several putative hydrolases predicts a typical α/β-hydrolase fold characteristic of lipolytic enzymes of the esterase/lipase/thioesterase family (EMBL-EBI InterPro search results, Figure 1). All of the lipases whose three-dimensional structures have been resolved so far exhibit this characteristic α/β fold [37]. The BLASTP tool was employed to find homologues of EstC among the non-redundant protein sequence data deposited at the National Center for Biotechnology Information database. BLASTP results show that EstC has very low identity outside putative hydrolases present among many S*treptomyces* members such as *S. sviceus* ATCC 29083 (EDY57684; 77% identity, 85% positive homology), *S. viridochromogenes* DSM 40736 (EFL34682; 77% identity, 84% positive homology) and *S. avermitilis* MA-4680 (NC_007333, 70% identity, 78% positive homology). However, in comparison to the functionally characterized intracellular esterases of *S. coelicolor* EstA and EstB, EstC shares no obvious sequence identity. Also, TCoffee alignments show that EstC shares little similarity with various members of the different bacterial lipase families. The closest similarity was found with members of group V, which comprises many cold-adapted lipases such as *Moraxella sp.* lipase 3 (P24640; 31% identity and 49% positive similarity) and *Psychrobacter* sp. G lipase (ADB11055; 31% identity and 51% positive similarity) [6], [38]. These results were confirmed with a BLAST against the Microbial Lipase and Esterase Database (MELDB), where EstC was assigned as a true lipase in a cluster with experimentally confirmed lipases of family V (MELDB cluster LiCl_6). Alignment with these lipases allowed the identification of the conserved catalytic triad formed by the pentapetide Gly-X_aa_-Ser-X_aa_-Gly, where the nucleophilic residue is Ser^116^ and the putative catalytic residues are Asp^241^ and His^268^ (Figure 1). However, the catalytic serine was found in a different motif GHSLGGQ (Gly^114^-Glu^120^) that seems conserved among streptomycetes. Finally, lipases generally retain the conserved N-terminal sequence HG, which was proposed to act as an oxyanion hole during catalysis [6], [39]. Our alignment shows the presence of an altered sequence PGFTG (Pro^46^-Gly^50^) that aligns with the family V oxyanion hole motif HGFGG (Figure 1).

**Figure 2:**
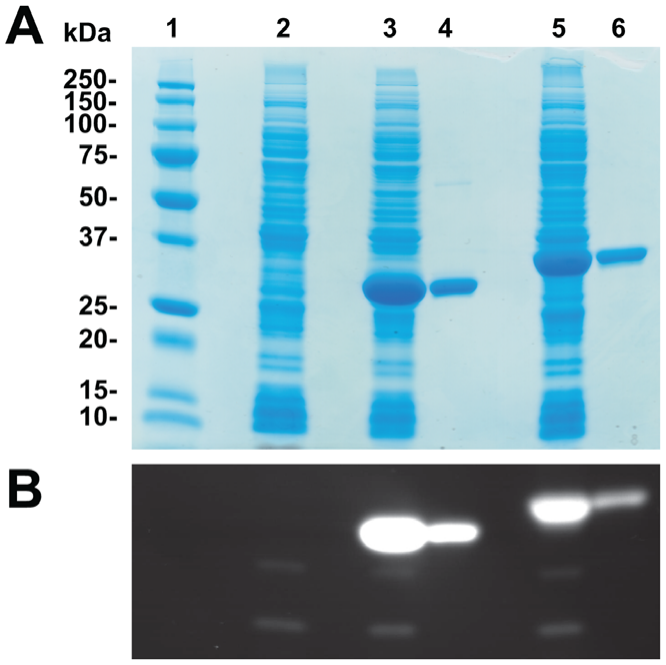
SDS-PAGE and zymogram of purified recombinant proteins. A) SDS-PAGE. Lane 1: Precision Plus All Blue molecular weight standard (Bio-Rad). Lane 2: Soluble fraction of control strain harboring vector pET16b. Lanes 3 and 5: Soluble fractions of *E. coli* strains harboring pET-EstB and pET-EstC vectors, respectively. Lane 4: Purified EstB after IMAC chromatography. Lane 6: Purified EstC after IMAC chromatography. B) Zymogram. Lane order is identical to panel A. The equivalent of 5 µg of purified enzymes were loaded in lanes 4 and 6.

The His-tagged recombinant EstC protein was purified to near homogeneity using a simple immobilized metal affinity chromatography (IMAC) (Figure 2). Two endogenous lipolytic enzymes were present in the *E. coli* strain with control plasmid, both of which were further efficiently discarded by chromatography (Figure 2B). The recombinant EstC protein showed an apparent molecular weight of 35 kDa, consistent with the expected theoretical weight of 34.6 kDa (including the His-tag). Silver-stained SDS-PAGE showed no protein contaminant and the identity of the protein was confirmed with Anti-His-tag Western blot analysis (results not shown). The identified protein was further digested for peptide sequencing using mass spectrometry with Mascot analysis. The peptide sequences identified by Mascot were solely attributed to the NP_629313 (EstC) protein of *S. coelicolor* A3(2), with an excellent 39% sequence coverage and MOWSE score of 400, providing unequivocal evidence of the purified protein identity. Mass spectrometry analysis of the purified protein confirmed a total protein mass of 34665 Da, corresponding exactly to the mass of the His-tagged EstC without its initiator formyl-methionine.

**Figure 3:**
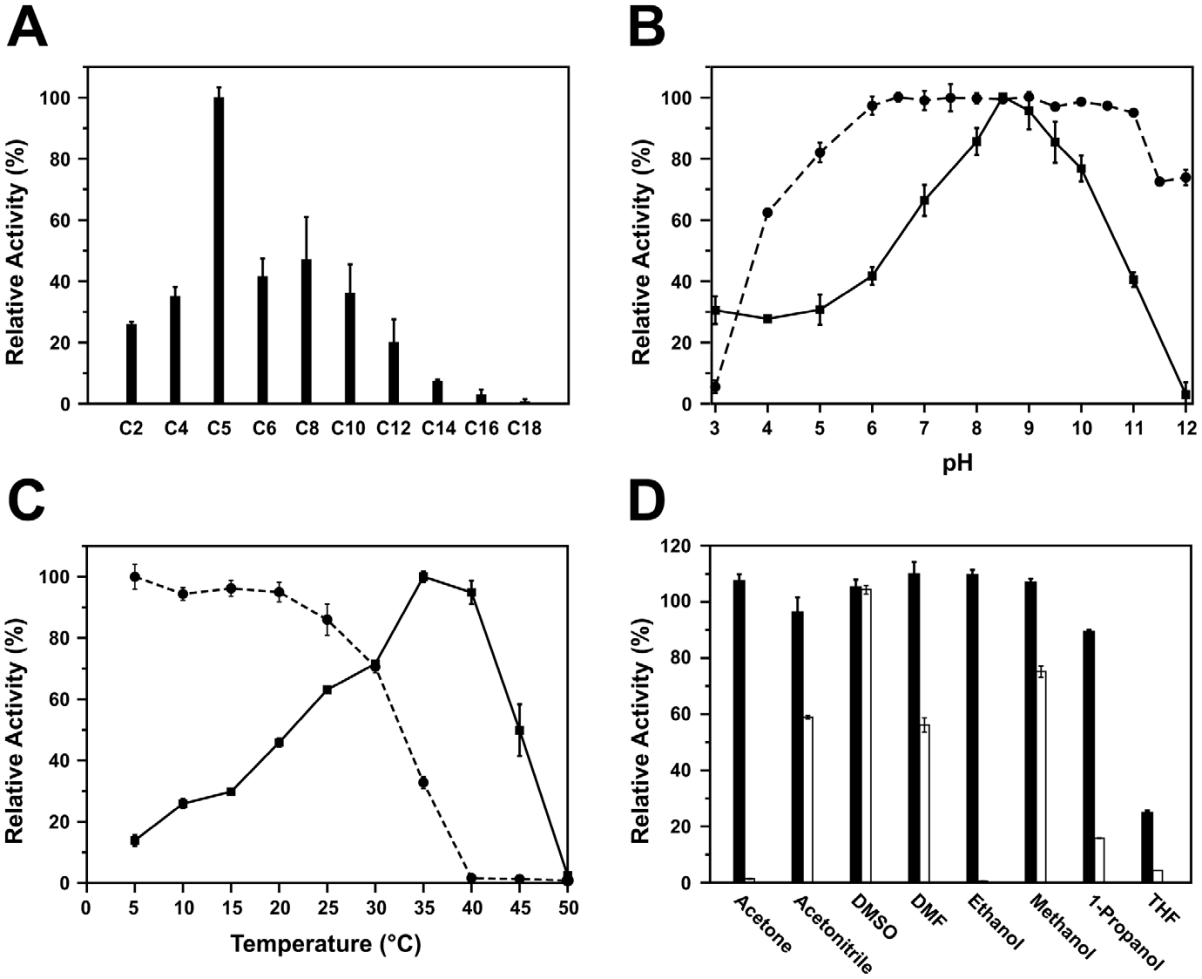
Effect of substrate, pH, temperature and organic solvents on EstC activity. A) Substrate discrimination by EstC. Enzyme activities are expressed relative to an optimal activity observed with substrate *p*NP-C5 (100% = 6.0±0.2 U/mg). B) Effect of pH on EstC activity. The solid line (squares) represents relative activity as a function of pH. The dashed line (circles) represents the residual activity after a 1-hour preincubation of the enzyme at various pH values. Enzyme activities are expressed relative to a maximal activity observed at pH 8.5. C) Effect of temperature on EstC activity. The solid line (squares) represents relative activity as a function of temperature. The dashed line (circles) represents the residual activity after a 1-hour preincubation of the enzyme at various temperatures. Values are expressed relative to a maximal activity observed at 35°C. D) Effect of organic solvents on EstC activity. Solid bars represent the residual activity after a 1-hour pre-incubation of the enzyme in 10% (v/v) of each organic solvent. Open bars represent residual activity after a 1-hour pre-incubation in 30% (v/v) of each organic solvent. Activities are calculated relative to a control enzyme incubated in the same conditions without organic solvent.

Substrate preference of the recombinant EstC was determined using various *p*-nitrophenyl esters (C2 to C18). EstC was primarily active toward short and medium chain esters (C2 to C12), with an optimal activity observed with *p*NP-C5 (Figure 3A). While the enzyme can hydrolyze long-chain esters, the measured activities are low relative to short-chain esters, showing only 7.4±0.5% and 2.9±1.7% for *p*NP-C14 and *p*NP-C16, respectively. Enzyme activity is practically nonexistent against *p*NP-C18. Cold-active lipase LipP from *Pseudomonas* sp. (strain B11-1) also showed similar short to mid-chain substrate specificity and very poor activity toward long chain substrates [29]. These results suggest that EstC could be classified as an intracellular esterase rather than a true lipase [40]. The optimal pH of EstC was tested using *p*NP-C8 in a pH range of 3–12. The enzyme was typically active at alkaline pH, with more than 75% of its maximal activity observed between pH 7.5 and 10 (Figure 3B). Still, maximal activity was observed in a very narrow range of pH 8.5–9. Nevertheless, the enzyme showed remarkable tolerance over a wide range of pH, with almost full residual activity observed between pH 6 and 11 (Figure 3B). Similar remarkable alkaline tolerance has also been reported on the *S. coelicolor* EstB and cold-active lipase LipA1 isolated from an Antarctic strain of *Psychrobacter* sp. 7195 [23], [41]. Alkaline tolerance of lipases is a very useful property for a number of industrial applications such as additives for laundry detergents.

The effect of temperature on EstC was determined by monitoring the hydrolysis of *p*NP-C8 at various temperatures from 5 to 50°C. Maximum activity was observed at 35–40°C but the enzyme retained more than 25% of its activity at 10°C (Figure 3C), suggesting it is also a cold-active esterase like EstB [23]. Similar temperature activity was also observed for the cold-active lipase of the mesophilic *Streptococcus* sp. N1 (optimal temperature of 37°C) and various cold-active lipases such as KB-Lip from *Pseudomonas* sp. (strain KB700A) and a lipase secreted by *Aspergillus nidulans* WG312 [30], [42]. Typically, enzymes from psychrophilic organisms display higher catalytic efficiencies between 0°C and 20°C rather than at mid-range or higher temperatures, but are susceptible to thermal denaturation even at medium temperatures (25–45°C) [39], [43]. Thermal stability of EstC was assayed with a pre-incubation period of 1 hour at various temperatures. The enzyme was relatively susceptible to thermal denaturation, showing a gradual loss of activity above 25°C and nearly no residual activity after 1 hour at only 40°C, suggesting it really is a cold-active enzyme (Figure 3C). These results show that EstC is more heat-labile than its *S. coelicolor* cytoplasmic counterpart EstB [23]. Nevertheless, the specific activity of EstC toward *p*NP-C5 was 4-fold higher at 10°C than the specific activity measured for EstB under the same conditions (Figure S1). These results suggest that EstC could be an interesting candidate for applications requiring cold conditions such as oil-contaminated soil bioremediation and biosynthesis of fragile pharmaceutical compounds.

Incubation with various water miscible organic solvents showed that EstC is remarkably tolerant to low concentrations of protein denaturants such as acetonitrile, dimethyl formamide (DMF), and primary alcohols (Figure 3D). Similar solvent tolerance was found with the cold-active LipP of *Pseudomonas* sp. (strain B11-1), but EstC showed greater tolerance to various concentrations of acetonitrile [29]. However, EstC was sensitive to tetrahydrofuran (THF) in both low (10% v/v) and medium concentration (30% v/v). The enzyme was also sensitive to all solvents in medium concentration, except for dimethyl sulfoxide (DMSO). In fact, pre-incubation of the enzyme with low concentrations (10% v/v) of acetone, DMF, DMSO, methanol and ethanol increased its activity, a phenomenon observed with many lipolytic enzymes such as LipP and a cold-active lipase from *Aeromonas* sp. LPB 4 [29], [44], [45]. The activation of EstC by DMSO and other organic solvents is an interesting feature for organic synthesis applications [46].

The effect of pre-incubation of various metal ions and chemicals on enzyme activity was also investigated (Table 1). The results showed no stimulation by any of the divalent cations tested. Ca^2+^ and Mg^2+^ had no significant impact on enzyme activity, but significant inhibition was observed with Cu^2+^, Fe^2+^ and Mn^2+^ at 10 mM concentration (Table 1). Co^2+^, Ni^2+^ and Zn^2+^ had a strong negative effect on enzyme activity at 1 mM, suggesting that these metals could be potent inhibitors. However, there were no effect on enzyme activity upon incubation with the metal chelators EDTA and EGTA, suggesting that EstC does not rely on any metal cofactor for activity. These results are in agreement with the fact that lipases and esterases generally do not require cofactors for catalysis [47]. Incubation with 10 mM DTT reduced enzyme activity by 40%, a sharp contrast relative to the activation effect reported for EstB in similar conditions [23]. The mature protein sequence of EstC contains only one cysteine residue, thus precluding the formation of any disulfide bridge, which is consistent with the relatively weak impact of DTT on enzyme activity. The absence of disulfide bridges is common among cold-active enzymes and could explain their inherent thermolability [28]. In contrast, the serine hydrolase inhibitor PMSF only reduced enzyme activity by 18% at 1 mM, showing that EstC is remarkably tolerant to this inhibitor. PMSF most likely binds to the catalytic serine nucleophile, confirming that EstC is a serine hydrolase like other lipolytic enzymes [3].

**Table 1:** Effect of metal and potent inhibitor concentrations on the activity of purified EstC.

Compound^a^	Concentration (mM)	Residual activity (% ± SD)^b^
Control	0	100±3
CaCl_2_	1	88±1
	10	92±3
CoCl_2_	1	56±1
	10	24±5
CuCl_2_	1	75±0.1
	10	26±4
FeCl_2_	1	83±0.3
	10	8±3
MgCl_2_	1	93±0.6
	10	96±3
MnCl_2_	1	88±1
	10	79±1
NiCl_2_	1	39±5
	10	10±0.2
ZnCl_2_	1	18±1
	10	7±0.1
EDTA	1	95±0.7
	10	98±3
EGTA	1	95±1
	10	94±3
DTT	1	92±2
	10	62±2
PMSF	1	82±0.3
	10	10±4

a All compounds were diluted in 50 mM Tris-HCl pH 8.5. An aliquot of the enzyme was incubated for 1 hour at 25°C with the buffer containing 1 or 10 mM of the compound. Values are expressed relative to a control without compound incubated in the same conditions.

b Residual activity was measured using *p*NP-C5 as substrate at a temperature of 35°C. The pre-incubated enzyme was diluted 10-fold before being used in standard assays. Residual activity was expressed relative to the activity measured with the control.

To evaluate the potential of EstC in ester synthesis, kinetic parameters of the enzyme were assessed with various short chain *p*NP-esters (C2 to C8) and TAGs (C2:0 and C4:0). At high substrate concentrations, both EstB and EstC showed clear substrate inhibition for all the *p*NP esters tested, results that directly correlated with the length of the acyl chain (results not shown). Increasing the surfactant concentration 5- and 10-fold did not reduce this phenomenon. As a result, kinetic parameters are reported for substrate concentrations where the enzyme displays Henri-Michaelis-Menten behavior. The kinetic parameters of EstC are summarized in Table 2. The *K*_m_ values decrease as the acyl chain length increases for substrates C2 to C8, with very similar values for *p*NP-C6 and *p*NP-C8. Our results show that EstC has the strongest affinity toward *p*NP-C6 (*K*_m_ = 0.071±0.004 mM). However, *k*_cat_ for this substrate is the lowest of the *p*NP esters tested. EstC displays much lower affinity for *p*NP-C2 than EstB (*K*_m_ = 0.89±0.06 mM) and EstA (*K*_m_ = 1.71±0.02 mM), but greater affinity toward C4 to C8 acyl chains [23], [24]. In fact, catalytic efficiency (*k*_cat_/*K*_m_) is highest toward *p*NP-C5 (737±77 mM^−1^ s^−1^), in agreement with the results obtained for substrate specificity (Figure 2A). The *k*_cat_ values of EstC toward short-chain *p*NP esters and triglycerides are comparable to those of the cold-active esterases of *Psychrobacter* sp. Ant300 and *Psychrobacter* sp. TA144 (formerly *Moraxella* sp. TA144) [48], [49]. These results suggest that EstC could be a very useful biocatalyst for the industrial synthesis of volatile short chain esters such as ethyl butyrate and ethyl valerate, two important flavor esters [50]. Enzyme specificity toward short-chain triglycerides was also assessed. In the conditions tested, EstC showed a very low affinity toward glyceryl triacetin (C2:0), with a very high *K*_m_ of 90±1 mM. To our knowledge, no such low affinity has ever been reported for cold-adapted esterases [28]. Nevertheless, *k*_cat_ toward this substrate was also very high, suggesting that EstC could be used at very high substrate concentrations for bioconversion of short chain triglycerides into valuable esters. It is well known that temperature can influence substrate affinity of an enzyme [29]. Quantification of the effect of temperature on the Michaelis constant of EstC could highlight better conditions to favor the enzyme affinity toward short chain triglycerides. Nevertheless, the enzyme showed much better affinity toward glyceryl tributyrin (C4:0), displaying a *K*_m_ value comparable to that of *Psy*HSL of *Psychrobacter* sp. Ant.300 (*K*_m_ = 0.115±0.30 mM) [49]. However, the relatively low *k*_cat_/*K*_m_ values of EstC toward short chain triglycerides suggest they might not be natural substrates of the enzyme.

**Table 2:** Kinetic parameters of recombinant EstC toward *p*-nitrophenyl short chain esters and triglycerides.

Substrate	*K*_m_	*k*_cat_	*k*_cat_ */K*_m_
	(mM)	(s^−1^)	(s^−1^ mM^−1^)
*p*-nitrophenyl acetate (C2)	2.9±0.8	451±10	156±41
*p*-nitrophenyl butyrate (C4)	0.84±0.05	124±2	148±10
*p*-nitrophenyl valerate (C5)	0.27±0.03	199±6	737±77
*p*-nitrophenyl hexanoate (C6)	0.071±0.004	41±1	578±36
*p*-nitrophenyl octanoate (C8)	0.11±0.018	58±3	527±90
Glyceryl triacetin (C2:0)	90±1	151±2	1.7±0.03
Glyceryl tributyrin (C4:0)	0.62±0.3	14±1	23±12

Standard deviation was derived from two different experiments, each performed with three replicates.

While EstC is not the most efficient lipolytic enzyme at very low temperatures, it still demonstrates unique features that deserve further consideration for cold-adapted industrial processes. The industrial use of cold-active esterases and lipases requires an easy and rapid production system that can generate very high yields of active enzyme in a short amount of time. Alas, most of the psychrophilic bacteria identified as sources of cold-active lipolytic hydrolases are slow growing and/or difficult to culture [28]. Additionally, industrial applications require high yield, economical and rapid purification steps that are easily amenable to the large-scale production of homogeneous and active biocatalysts. In addition to being inherently thermolabile, lipases and esterases from cold-adapted bacteria are notoriously difficult to purify due to the production of lipopolysaccharides that strongly associate with these lipolytic enzymes [28]. To overcome these issues, we developed an *E. coli* recombinant system that can achieve high yields of EstC production from simple LB medium growth at low temperature in only 24 hours. Under these conditions, the enzyme was overproduced as a cytoplasmic, soluble biocatalyst with yields exceeding 350 mg/L. Additionally, EstC was easily purified to near homogeneity from a fast, single-step IMAC with an average yield of 42.8±2.91 mg (average of three independent lots), confirming that the enzyme is an attractive candidate for large-scale production. This represents a considerable advantage over existing cold-active lipolytic enzymes isolated from mesophilic and psychrophilic organisms, the latter having failed to be purified to homogeneity to date [28]. As a result, EstC currently represents one of the fastest, easiest, and most efficient alternatives for the industrial production of cold-active esterases with high yields and high purity. Additionally, since EstC was isolated from a mesophilic organism, it remains relatively active and thermostable at higher temperatures, retaining more than 70% of its residual activity at temperatures up to 30°C. This represents a major advantage over lipolytic enzymes isolated from cold-adapted organisms [28]. Finally, high yields of intracellular EstC expression could provide a strong basis for the development of whole-cell industrial biocatalysts, potentially eliminating the need for cell lysis and purification steps altogether. A similar approach was recently developed by Wei and coworkers on a lipase from *Proteus* sp., whereby the enzyme was used as an efficient catalyst directly from the overexpressed *E. coli* BL21 (DE3) cells [51]. In this study, the recombinant cells were simply permeabilized with a detergent and used as whole-cell catalysts for the conversion of oils into biodiesel. We are currently investigating the feasibility of such an approach applied to the cold-active EstC.

### Conclusion

Genome mining of intracellular lipolytic enzymes in *S. coelicolor* has led to the preliminary characterization of the gene product of SCO5165, a new cold-active esterase named EstC. Our results demonstrate that this enzyme is tolerant to alkaline pH and effective at medium to low temperatures. In agreement with the previously characterized intracellular esterase EstB, EstC shows good alcohol and solvent tolerance, in addition to displaying enhanced activity after pre-incubation with low concentrations of organic solvents. The enzyme also exhibits a higher specificity for shorter chain *p*NP esters (C4 to C8), suggesting it could be a serious candidate for ester synthesis in organic media. This further highlights the industrial potential of EstC for various biotechnological applications, an approach that we are currently investigating.

## Supporting Information

Figure S1 **Specific activities of EstB and EstC toward** ***p*** **NP-C5 at different temperatures.**
(TIF)

Table S1 **Primers employed for amplification of ORFs coding for putative non-secreted lipolytic enzymes of** ***S. coelicolor*** **A3(2).**
(DOC)

## References

[1] Sharma D, Sharma B, Shukla AK (2011). Biotechnological approach to microbial lipase: a review.. Biotechnology.

[2] Sharma R, Chisti Y, Banerjee UC (2001). Production, purification, characterization, and applications of lipases.. Biotechnol Adv.

[3] Jaeger KE, Dijkstra BW, Reetz MT (1999). Bacterial biocatalysts: molecular biology, three-dimensional structures, and biotechnological applications of lipases.. Annu Rev Microbiol.

[4] Gupta R, Gupta N, Rathi P (2004). Bacterial lipases: an overview of production, purification and biochemical properties.. Appl Microbiol Biotechnol.

[5] Jaeger KE, Eggert T (2002). Lipases for biotechnology.. Curr Opin Biotechnol.

[6] Arpigny JL, Jaeger KE (1999). Bacterial lipolytic enzymes: classification and properties.. Biochem J.

[7] Verger R (1997). “Interfacial activation” of lipases: facts and artifacts.. Tibtech.

[8] Fojan P, Jonson PH, Petersen MT, Petersen SB (2000). What distinguishes an esterase from a lipase: a novel structural approach.. Biochimie.

[9] Uppenberg J, Hansen MT, Patkar S, Jones TA (1994). The sequence crystal structure determination and refinements of two crystal forms of lipase B of *Candida antarctica.*. Structure.

[10] Szatjer H, Maliszewska I, Wieczorek J (1988). Production of exogenous lipases by bacteria, fungi and actinomycetes.. Enzyme Microb Technol.

[11] Gandolfi R, Marinelli F, Lazzarini A, Molinari F (2000). Cell-bound and extracellular carboxylesterases from *Streptomyces*: hydrolytic and synthetic activities.. J Appl Microbiol.

[12] Large KP, Mirjalili M, Osborne M, Peacock LM, Zormpaidis V (1999). Lipase activity in streptomycetes.. Enzyme Microb Technol.

[13] Sommer P, Bormann C, Gotz F (1997). Genetic and biochemical characterization of a new extracellular lipase from *Streptomyces cinnamomeus.*. Appl Environ Microbiol.

[14] Vujaklija D, Schroder W, Abramic M, Zou P, Lescic I (2002). A novel streptomycete lipase: cloning, sequencing and high-level expression of the *Streptomyces rimosus* GDS(L)-lipase gene.. Arch Microbiol.

[15] Shim M, Kim W, Kim J (1997). Neutral lipids and lipase activity for actinorhodin biosynthesis of *Streptomyces coelicolor* A3(2).. Biotechnol Lett.

[16] Arabolaza A, Rodriguez E, Altabe S, Alvarez H, Gramajo H (2008). Multiple pathways for triacylglycerol biosynthesis in *Streptomyces coelicolor.*. Appl Environ Microbiol.

[17] Alvarez HM, Steinbuchel A (2002). Triacylglycerols in prokaryotic microorganisms.. Appl Microbiol Biotechnol.

[18] Popova NI, Krivova AY, Rastimeshina IO, Burtseva SA (2005). Interrelation of the biosyntheses of lipids, lipoxygenase, and lipase in cultured streptomycetes.. Microbiology.

[19] Olukoshi ER, Packter NM (1994). Importance of stored triacylglycerols in *Streptomyces*: possible carbon source for antibiotics.. Microbiology.

[20] Schauner C, Dary A, Lebrihi A, Leblond P, Decaris B (1999). Modulation of lipid metabolism and spiramycin biosynthesis in *Streptomyces ambofaciens* unstable mutants.. Appl Environ Microbiol.

[21] Bielen A, Cetkovic H, Long PF, Schwab H, Abramic M (2009). The SGNH-hydrolase of *Streptomyces coelicolor* has (aryl)esterase and a true lipase activity.. Biochimie.

[22] Bentley SD, Chater KF, Cerdeno-Tarraga AM, Challis GL, Thomson NR (2002). Complete genome sequence of the model actinomycete *Streptomyces coelicolor* A3(2).. Nature.

[23] Soror SH, Verma V, Rao R, Rasool S, Koul S (2007). A cold-active esterase of *Streptomyces coelicolor* A3(2): from genome sequence to enzyme activity.. J Ind Microbiol Biotechnol.

[24] Soror SH, Rao R, Cullum J (2009). Mining the genome sequence for novel enzyme activity: characterisation of an unusual member of the hormone-sensitive lipase family of esterases from the genome of *Streptomyces coelicolor* A3 (2).. Protein Eng Des Sel.

[25] Coté A, Shareck F (2008). Cloning, purification and characterization of two lipases from *Streptomyces coelicolor* A3(2).. Enzyme Microb Technol.

[26] Kieser T, Bibb MJ, Buttner MJ, Chater KF, Hopwood DA (2000). Pratical *Streptomyces* genetics.

[27] Diaz P, Prim N, Javier Pastori FI (1999). Direct fluorescence-based lipase activity assay.. Biotechniques.

[28] Joseph B, Ramteke PW, Thomas G (2008). Cold active microbial lipases: some hot issues and recent developments.. Biotechnol Adv.

[29] Choo DW, Kurihara T, Suzuki T, Soda K, Esaki N (1998). A cold-adapted lipase of an Alaskan psychrotroph, *Pseudomonas* sp. strain B11-1: gene cloning and enzyme purification and characterization.. Appl Environ Microbiol.

[30] Rashid N, Shimada Y, Ezaki S, Atomi H, Imanaka T (2001). Low-temperature lipase from psychrotrophic *Pseudomonas* sp. strain KB700A.. Appl Environ Microbiol.

[31] Alquati C, Gioia LD, Santarossa G, Alberghina L, Fantucci P (2002). The cold-active lipase of *Pseudomonas fragi*: heterologous expression, biochemical characterization and molecular modeling.. Eur J Biochem.

[32] Jeon JH, Kim JT, Kim YJ, Kim HY, Lee HS (2009). Cloning and characterization of a new cold-active lipase from a deep-sea sediment metagenome.. Appl Microbiol Biotechnol.

[33] Para LP, Reyes F, Acevedo JP, Salazar O, Andrews BA (2008). Cloning and fusion expression of a cold-active lipase from marine Antarctic origin.. Enzyme Microb Technol.

[34] Ryu HS, Kim HK, Choi WC, Kim MH, Park SY (2006). New cold-adapted lipase from *Photobacterium lipolyticum* sp. nov. that is closely related to filamentous fungal lipases.. Appl Microbiol Biotechnol.

[35] Yang XX, Lin XZ, Fan TJ, Bian J, Huang XH (2004). Cloning and expression of *lip*P, a gene encoding a cold-adapted lipase from *Moritella* sp. 2-5-10-1.. Curr Microbiol.

[36] Zhang P, Zeng R (2007). Cloning, expression, and characterization of a cold-adapted lipase gene from an Antarctic deep-sea psychrotrophic bacterium.. J Microb Biotechnol.

[37] Ollis DL, Cheah E, Cygler M, Dijkstra B, Frolow F (1992). The alpha/beta hydrolase fold.. Protein Eng.

[38] Xuezheng L, Shuoshuo C, Guoying X, Shuai W, Ning D (2010). Cloning and heterologous expression of two cold-active lipases from the Antarctic bacterium *Psychrobacter* sp. G.. Polar Res.

[39] Roh C, Villatte F (2008). Isolation of a low-temperature adapted lipolytic enzyme from uncultivated micro-organism.. J Appl Microbiol.

[40] Kim HK, Jung Y, Choi W, Ryu HS, Oh T (2004). Sequence-based approach to finding functional lipases from microbial genome databases.. FEMS Microbiol Lett.

[41] Zhang P, Zeng R (2006). Cloning, expression, and characterization of a cold-adapted lipase gene from an Antarctic deep-sea psychrotrophic bacterium, *Psychrobacter* sp. 7195.. J Microb Biotechnol.

[42] Mayordomo I, Randez-Gil F, Prieto JA (2000). Isolation, purification, and characterization of a cold-active lipase from *Aspergillus nidulans.*. J Agric Food Chem.

[43] Arpigny JL, Feller G, Gerday C (1993). Cloning, sequence and structural features of a lipase from the antarctic facultative psychrophile *Psychrobacter immobilis* B10.. Biochim Biophys Acta.

[44] Lee MY, Dordick JS (2002). Enzyme activation for nonaqueous media.. Curr Opin Biotechnol.

[45] Lee HK, Ahn MJ, Kwak SH, Song WH, Jeong BC (2003). Purification and characterization of cold active lipase from psychrotrophic *Aeromonas* LPB 4.. J Microbiol.

[46] Klibanov AM (2001). Improving enzymes by using them in organic solvents.. Nature.

[47] Jaeger KE, Reetz MT (1998). Microbial lipases form versatile tools for biotechnology.. Trends Biotechnol.

[48] Kulakova L, Galkin A, Nakamaya T, Nishino T, Esaki N (2004). Cold-active esterase from *Psychrobacter* sp. Ant300: gene cloning, characterization, and the effect of Gly→Pro substitution near the active site on its catalytic activity and stability.. Biochim Biophys Acta.

[49] De Santi C, Tutino ML, Mandrich L, Giuliani M, Parrilli E (2010). The hormone-sensitive lipase from *Psychrobacter* sp. TA144: new insight in the structural/functional characterization.. Biochimie.

[50] Alvarez-Macarie E, Baratti J (2000). Short chain flavour ester synthesis by a new esterase from *Bacillus licheniformis.*. J Mol Catal B Enzym.

[51] Gao B, Su E, Lin J, Jiang Z, Ma Y (2009). Development of recombinant *Escherichia coli* whole-cell biocatalyst expressing a novel alkaline lipase-coding gene from *Proteus* sp. for biodiesel production.. J Biotechnol.

[52] Notredame C, Higgins DG, Heringa J (2000). T-Coffee: A novel method for fast and accurate multiple sequence alignment.. J Mol Biol.

[53] Poirot O, O'Toole E, Notredame C (2003). Tcoffee@igs: A web server for computing, evaluating and combining multiple sequence alignments.. Nucleic Acids Res.

