# Supplementary figures and images for: Isolation and Characterization of EstC, a New Cold-Active Esterase from Streptomyces coelicolor A3(2)
Source: PLoS One. 2012 Mar 2;7(3):e32041. doi: 10.1371/journal.pone.0032041 (PMC3292560; doi:10.1371/journal.pone.0032041)

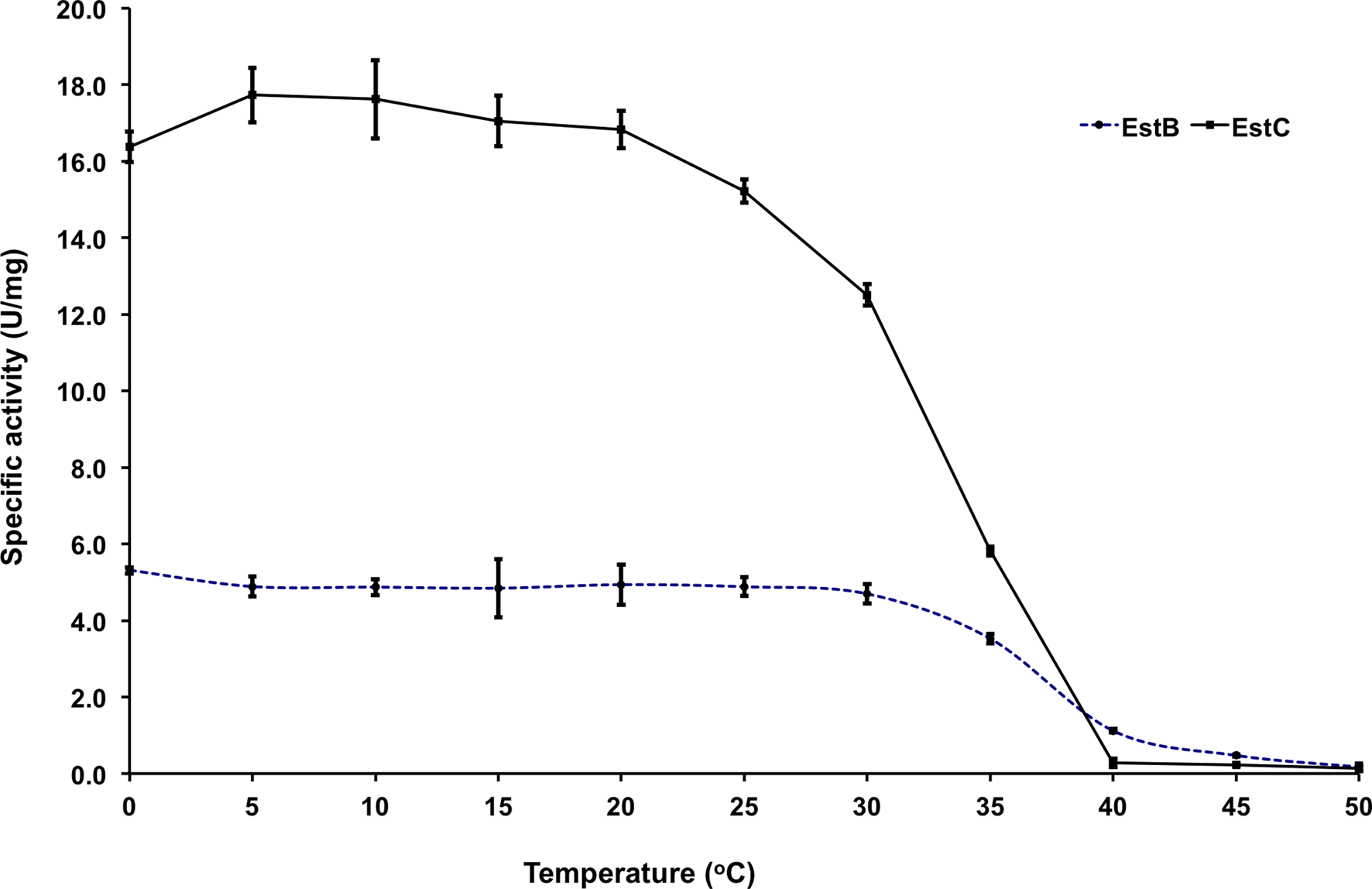

Supplement: Figure S1 — Specific activities of EstB and EstC toward p NP-C5 at different temperatures. (TIF) [file pone.0032041.s001.tif]
