# Supplementary material for: Isolation and Characterization of EstC, a New Cold-Active Esterase from Streptomyces coelicolor A3(2)
Source: PLoS One. 2012 Mar 2;7(3):e32041. doi: 10.1371/journal.pone.0032041 (PMC3292560; doi:10.1371/journal.pone.0032041)
Supplement: Table S1 — Primers employed for amplification of ORFs coding for putative non-secreted lipolytic enzymes of S. coelicolor A3(2). (DOC) [file pone.0032041.s002.doc]

| Gene | Accession | 5’ primer (*Nde*I) | 3’ primer (*Xho*I) |
| --- | --- | --- | --- |
|  | number |  |  |
| SCO1265 | NP_625552 | AAACATATGAGTTTCCTCAATCCC | ATTCTCGAGTCAGTCGGCCAGGG |
|  |  | CGTATCCCAG | CCTTGAGGACTTC |
| SCO2123 | NP_626380 | ATTCATATGTCGGTCCTGCCCGGA | ATTCTCGAGTCAGCCACCTGCGGC |
|  |  | GCCGAGCC | CGTCCCTTC |
| SCO3644 | NP_627838 | AAACATATGCCGGACGCCGCCGC | AAACTCGAGTCACCAGGCCAACT |
|  |  | AGAACC | GCGCGATCTC |
| SCO4746 | NP_628904 | AAACATATGCACGTGAGCGAGAG | AATCTCGAGTCAGCCGGGCCCTG |
|  |  | CAACG | TGCTGCTGG |
| SCO5165 (*estC*) | NP_629313 | AAACATATGGTGAGCAGGAACGC | TTTCTCGAGTCAGCGCACGTACG |
|  |  | CGCCTTC |  |
| SCO6967 (*estB*) | NP_631032 | AAACATATGGCCGAGGCCCGCGA | ATTCTCGAGTCAGCGGGCGAGCA |
|  |  | GCACACG | CGCCGTC |
| SCO7131 (*estA*) | NP_631192 | AAACATATGAGCGACATCGTTCT | ATACTCGAGTCAGTCGGTGCCGA |
|  |  | CGAACC | GGGCACTG |
